# Feelings of older Japanese primiparous couples and satisfaction of older primiparous wives with their husbands’ support during pregnancy: Focus on the perceptions of pregnant couples

**DOI:** 10.1002/nop2.509

**Published:** 2020-06-30

**Authors:** Kumiko Nakajima, Atsumi Usui, Yuko Hayakawa

**Affiliations:** ^1^ School of Nursing Faculty of Health Science Gunma Paz University Takasaki Japan

**Keywords:** family therapy, mental health promotion, social support and qualitative methodology

## Abstract

**Aim:**

This study was to explore the feelings of older primiparous wives and husbands and satisfaction of older primiparous wives with their husbands’ support during pregnancy.

**Design:**

This study is a qualitative research design based on the characteristics of clarifying the recognition of older primiparous couples.

**Methods:**

Participants were eight older Japanese primiparous couples. Older primiparous couple's feeling and support by husband during pregnancy were elected using semi‐structured interviews. The analysis was used by content analysis method.

**Results:**

Two categories of the couples' feeling during pregnancy included “Mental stress and physical burden associated with older age” and “Richness and strong will to actively accept the older age.” Three categories of the husbands’ support for wives’ satisfaction included “Empathy regarding the older primiparous wives,” “physical and mental health” and “Cooperation of housework with husband.” This study has greatly contributed to nursing support for marital relationships.

## 1 INTRODUCTION

The number of older primiparas (aged ≥35 years) is increasing in Japan. The percentage of first births among total live births to women aged ≥35 years rose from 4.7% in 2004–9.5% in 2013 (Maehara et al., 2016). Some studies have claimed that older primiparas are exposed to higher risks during pregnancy and delivery. In addition, older primiparas often face more physical burdens after delivery (Sugiura, 2013). Compared with younger primiparas and multiparas, older primiparas face higher levels of anxiety about child‐rearing and postpartum depression (Mori et al., 2016). Older primiparas may therefore demand more careful consideration from healthcare professionals not only after childbirth, but also during pregnancy.

On another hand, according to precedent research investigating experiences and thoughts at 1 month after delivery, older primiparas had strong mental and social characteristics supported by their life experience (Niimura & Ogawa, 2012). Older primiparous women also display a unique strength derived from gratitude for a safe birth and becoming a mother, flexibility in their thinking, a greater ability to take care of themselves and a greater potential for effective parenting (Mori et al., 2014; Sakajo et al., 2014). It is therefore necessary to understand and support the mental and social characteristics of older primiparas.

Maehara et al. (2014) reported that emotional support was one of the factors positively associated with increased confidence as mothers among older primiparas at 1 month postpartum. By contrast, a lack of communication with her partner about the parenting role was negatively associated with maternal confidence. That is, to promote confidence in the motherhood role of older primiparas after birth, receiving support from a husband is thought to be important. When considering nursing support for older primiparous couples, clarifying the feelings of older primiparas and their husbands is important.

## 2 BACKGROUND

In recent years, general support for pregnant women has decreased in concurrence with the growth of nuclear families and urbanization. Therefore, support from husbands has become increasingly important. We reported that support from husbands is important for their wives’ satisfaction during pregnancy (Nakajima, 2006). We also identified the following three concepts as perceptions about the husbands’ support for their wives’ satisfaction during pregnancy: “intimacy of the couple,” “family system” and “consciousness of parenthood” (Nakajima & Tokiwa, 2011). In addition, we suggested that the primiparous couple's feelings during pregnancy have common and different perceptions between husbands and wives from the viewpoint of the wives’ satisfaction with their husbands’ support, although it is important that both wives and husbands mutually understand their responsibilities about support and involvement in the marital relationship.

However, these studies do not give an adequate account of perception about older primiparous wives’ and husbands’ feelings during pregnancy or older primiparous wives’ satisfaction with their husbands’ support. Marital satisfaction and the harmonious relationship between a pregnant husband and wife can have a positive effect on postnatal marital relationships. Thus, the purpose of this study was to explore the feelings of older primiparous wives and husbands and satisfaction of older primiparous wives with their husbands’ support during pregnancy.

## 3 OPERATIONAL DEFINITIONS OF KEY TERMS

1. Older primiparous couples: first‐time pregnant women over 35 and their husbands.
2. Wife satisfaction: a feeling of happiness, relief on the part of the wife or feelings of being supported by her husband (Nakajima & Tokiwa, 2011).
3. Husband's supportiveness: supportive words, deeds, attitudes, emotions and intentions on the part of the husband (Nakajima & Tokiwa, 2008).

## 4 THE STUDY

### 4.1 Design

This study is a qualitative research design based on the characteristics of clarifying the recognition of older primiparous couples during pregnancy.

### 4.2 Methods

#### 4.2.1 Data collection

A midwife researcher with a PhD interviewed. Researchers have been involved in qualitative research for a long time and have extensive interview experience. Before the start of the study, there was no relationship between the researcher and the participants and interviews were conducted without knowing the participants' personal information.

The present qualitative study was part of a larger longitudinal study of Japanese older primiparas. Data were collected three times: pregnancy, 1 month and 3 months after childbirth. In this study, we used data from pregnancy.

An interview guide and a demographic guide were created with reference to a study (Nakajima & Tokiwa, 2011) that examined the support of husbands who satisfied with their wives for primiparas. Older primiparous couples’ feeling and support by husband during pregnancy were elected using semi‐structured interviews. Interviews were conducted with wives and husbands to talk separately, and we asked the wives: (a) How to accept the old pregnancy and the older primiparous wives’ feeling; (b) how to perceive of mental health and physical burden of the pregnancy; and (c) the situation and scene of husbands’ supportiveness for their wives’ satisfaction. In addition, we asked the husband: (a) How to perceive his wives’ old pregnancy and his feeling for the older primiparas; (b) how to perceive wives’ mental health and physical burden of the pregnancy; and (c) how to perceive the husbands’ supportiveness for his wife perceived as satisfactory.

These interviews were audio‐recorded and transcribed with the couples’ consent. And field notes were made during the interview. Interviews were conducted once for late pregnancy couples (range = 32–36 weeks). Each interview lasted an average of wives 37 min and husbands 34 min (19–70 min; husbands and wives were interviewed separately). The interviews were conducted at the couples’ appointed place or the researcher's university. The interviews were conducted during July–November 2016.

#### 4.2.2 Research participants

Qualitative studies describing the experience of old primiparas (Nelson., 2004) and the perceptions of primiparous couples (Nakajima & Tokiwa, 2011) were reported. In a qualitative study by Nelson, the sample size was seven older primiparas aged 35 and over and a phenomenological/interpretive approach was used to determine the meaning of age. According to a qualitative study of primiparous couples by Nakajima et al., a semi‐structured interview was conducted with eight couples, demonstrating the couples’ awareness about husbands’ support for their wives’ satisfaction during the early phase of pregnancy. The findings of those two studies were rich enough and covered enough of the dimensions.

The selection of the participants is as follows. In primiparous wives who were ≥35 years of age, normal pregnancy was included. The couples were able to communicate in Japanese. Pregnant wives and her infant with serious health problem were excluded.

The principal investigator and co‐researchers explained the purpose of the study to the older primiparous wives who participated in a maternity class by two maternity hospitals in two cities of north Kanto in Japan and requested research cooperation. The husbands were asked to cooperate in research through their wives. The participants in this study were couples who could cooperate.

Sixteen older primiparous couples were asked to investigate originally, but eight couples were excluded due to the lack of interview cooperation from the husband's job. Eight older primiparous couples were ultimately selected. Interviews were conducted with eight primiparous Japanese couples.

### 4.3 Analytical methods

Content analysis method (Sandelowski, 2000) was used for each member of both couples. The interviews were transcribed. A sentence was defined as including a subject and predicate and narrated by subjects concerning the husband's supportiveness perceived to be satisfactory by the wife. One data unit was defined as a context unit, which was also defined as a recording unit. To analyse partners’ perceptions of each other, we compared the recording units of a husband's supportiveness perceived by his wife and a husband's perception of his supportiveness towards his wife. We classified these units into common perceptions by husbands and wives and different perceptions between husbands and wives (wife‐only and husband‐only perceptions).

To ensure the reliability of the data, we tried to ensure the reliability of the data by directly confirming the content of the narrative obtained from the target couple. Three researchers who specialize in maternity nursing reproduced the results, and the results were ensured. Moreover, to increase the precision of categorical classifications, the categorical classification and selection of category names were repeated using the classification results and comments of the three researchers as a reference. There were no new data, no new coding and no new categories for the first seven couples, and even after analysing a complete sample of participants from eight couples, no new concepts were formed. As a result, the data reached saturation. The study was conducted according to an integrated standard for reporting qualitative studies (see File S1).

### 4.4 Ethical considerations

Participation in the study was voluntary. All couples were provided written and verbal assurance that refusal or exclusion from the study would not affect their medical services. Consent was obtained after couples had read and understood these terms. This study was approved by the Clinical Research Ethical Review Board of Gunma PAZ University (approval number PAZ16‐17).

## 5 RESULTS

### 5.1 Participant attributes

Table 1 shows the participants’ characteristics.

**TABLE 1:** Demographic and clinical characteristics (*N* = 8)

Item	Mean ± *SD* (range)	*N*
		
Wife's age	38.8 ± *SD* (35–47)	
Wife's employment		
Full time		3
Part time		2
Unemployed		3
Establishment of the pregnancy		
Natural pregnancy		6
Fertility treatment		2
Pregnancy progress		
Regular progress		6
Complication during pregnancy		2
Husband's age	42.8 ± *SD* (35–55)	
Husband's employment		
Full time		8
Family structure		
Nuclear family		8

### 5.2 The wives’ and husbands’ feelings during pregnancy

The feelings of an older primiparous wife and husband were labelled with 68 wives and 24 husband units, 93 codes, 13 subcategories and two categories (Table 2). The following is a summary of the results for each category. The features of category are explained by unit.

**TABLE 2:** Categories and subcategories of couples' perception of feeling during pregnancy

Categories	Couples' perceptions	Subcategories	Wives' perception	Husbands' perception
			Case	Case
1. Mental stress and physical burden associated with older age	Common perceptions of couples	Impatience quickly because of older age, wishing to become children	C, F	B, C, E, G
	Common perceptions of couples	Anxiety about the fetus congenital disease in older age	F, G	B, C
	Wives' perception	Weak‐minded feeling for older compared with younger primiparas	A, B, D	
	Wives' perception	Anxiety about the physical risk of being an older primipara	A, C, D, F, G	
	Wives' perception	Stress to the minor problems during pregnancy	A, C, D, E, F	
	Wives' perception	Mental stress about the physical burden of both pregnancy and work	B, C, E	
2. Richness and strong will to actively accept the older age	Common perceptions of couples	Regular pregnancy progress in spite of being an older primiparous wife	D, E, G, H	A, E, F, G
	Common perceptions of couples	Emotional ability to accept the older age positively	B, E, F, G, H	E, F, G
	Wives' perception	Preparedness to have a child in older age	A, B, D	
	Wives' perception	Feeling of doing well enough when surrounded by older primiparous women	A, E	
	Wives' perception	Feel the need to protect the foetus after assisted reproductive technology	A, E	
	Wives' perception	Decision to continue both the pregnancy and work by the wife	B, C, G, H	
	Husband's perception	Expectation for the motherhood that child mental will be healthy		D, H

#### 5.2.1 Category 1: Mental stress and physical burden associated with older age

Two subcategories represented the common perceptions shared between the husband and wife, such as impatience about being at an advanced age and wanting a child and uneasiness about foetal congenital disease. The main remarks are as follows:

I was anxious about fetal congenital disease. But I was able to believe in my baby when I saw the echo image. (wife G). The probability of Down syndrome increases with age. I did not tell my wife, but I am investigating it on the Internet (husband G).

Four subcategories represented wife‐only perceptions. Compared with younger pregnant women, older primiparas report feeling more insecure about physical risks, and feeling more of a mental burden because of minor troubling symptoms with their older age. Moreover, older primiparas feel increased physical and mental burdens in pregnancy and work. They remarked as follows:

I wish I had a baby when I was younger. I am thinking about the future of my child. I will be 40 years old when my child enters kindergarten (wife B).

I was told about gestational diabetes at a checkup in the early stage of pregnancy. Until that time, I did not have diabetes. I then realized that age really affected me (wife H).

#### 5.2.2 Category 2: Richness and strong will to actively accept the older age

Two subcategories represented the common perceptions between the husband and wife. The husband and wife realized that despite the advanced age of the wife, the pregnancy was making relatively favourable progress; in addition, the wife's advanced age had led to her mental maturity:

I have a lot of life experience because I am older, so I have emotional ability if anything happens, I didn’t get into a panic. I am anxious, but I am thinking that I enjoy my pregnancy (wife E). Since my wife has a lot of life experience compared with young people, I think she also has more knowledge and mental stability (husband E).

Four subcategories represented wife‐only perceptions. Older primiparas view their own advanced age positively rather than negatively. She said that that she could do so by seeing the surrounding older pregnant women being adapted to pregnancy. In addition, women who become pregnant after infertility treatment strongly desire to protect the life of the unborn baby. She was pleased that the husband was looking forward to the birth more than her. Moreover, she thought about her career and decided to continue the pregnancy:

I am surrounded by other pregnant women over 35 years of age, so I feel like I can do it well enough. If no older primiparas were around me, I think I would become anxious (wife E).

In my idea, the woman thinks that it is better to work during pregnancy. Therefore, it was important for me to decide on my own to continue work (wife B).

### 5.3 Husband's support for wife's satisfaction during pregnancy

Couple perceptions of husbands’ support during pregnancy were labelled with 80 wives’ units and 92 husbands’ units, 171 codes, 14 subcategories and three categories (Table 3). The following is a summary of the results for each category. The features of category are explained by unit.

**TABLE 3:** Categories and subcategories of couples' perception of husbands' support for the satisfaction of wives

Categories	Couples' perceptions	Subcategories	Wives' perception	Husbands' perception
			Case	Case
1. Empathy regarding the older primiparous wives’ physical and mental health	Common perceptions of couples	Empathy regarding the risk of mother and unborn baby by the older pregnancy	C, H	C, E, H
	Common perceptions of couples	Empathy for the minor trouble and fatigue of the pregnancy	A, B, C, D, E, G	A, B, C, D, E, F, G
	Common perceptions of couples	Empathy for the mental stress and physical burden to both of the pregnancy and the work	B, H	B, C
	Common perceptions of couples	Selecting the childbirth place and joining childbirth as his wishes to the anxiety about the old primiparous wives	B, C, D, E, H	C, E, G, H
	Husband's perception	Empathy for the wives' anxiety about older age		A, C, G
	Husband's perception	Anxiety about physical burden for the delivery and child‐rearing		E, F, H
	Husband's perception	Increase motivation for childcare support caring for wife after childbirth		C, E, F
2. Cooperation of housework with husband	Common perceptions of couples	Share of the housework before the pregnant wife	B, C, E, F, H	A, B, C, E, H
	Common perceptions of couples	Support with housework to show empathy for the pregnant wife	A, C, D, G	A, B, E, F, G
3. Adequate communication and sharing of thoughts between the couple in preparation for parenthood	Common perceptions of couples	Looking forward to the baby being born with the couple	B, C, D, E, F, G, H	D, G, H
	Common perceptions of couples	Communication and sharing of thoughts between the couple in preparation for childbirth and taking care of baby	A, B, C, E	A, B, F, G, H
	Common perceptions of couples	Communication between the couple in relation to childbirth	B, D, F, G, H	B, G
	Common perceptions of couples	Communication between the couple in relation to increasing amounts of housework and taking care of the baby after birth	B, C, F, G, H	C, F
	Common perceptions of couples	Communication between the couple in relation to the adjustment of schedule for child‐rearing and returning to work	C, D, F	B, C, D, F

#### 5.3.1 Categories 1: Empathy towards older primiparous wives’ physical and mental health

Four subcategories represented the common perceptions shared between the husband and wife. The husband was anxious about the physical and mental burdens accompanying the older primiparous wife's pregnancy. They mutually recognized the husband's consideration for the wife's satisfaction:

When I found out about gestational diabetes, my husband was worried and consulted with his mother. I found out later that my husband had been very worried about me (wife H). Although she has gestational diabetes, we usually eat together. Since my wife has observe dietary restrictions, I tell her, “You are great” (husband H).

At the time of childbirth, I think that I want my husband to be by my side. But he does not like to see blood. Therefore, I said, “Let's decide mutually at that time” (wife E). I hold my wife's hand and encourage her during childbirth. Since I do not like to see blood, I communicate through the midwife. To help my wife relax is my greatest purpose of attending the birth (husband E).

Three subcategories represented husband‐only perceptions about the husband and wife. The husband cared about the wife's being uneasy feeling about her older age in terms of childbirth and child‐rearing. The husband also had a desire to support child‐rearing after the birth:

I feel that I would also like to carry out child‐rearing together with my wife. Therefore, I think that I make myself like to be able to make general child‐rearing all (husband C).

#### 5.3.2 Category 2: Cooperation of housework with husband

Two subcategories represented the common perceptions shared between the husband and wife (Table 3). They mutually recognized the husband's support with housework for the wife's satisfaction:

My husband has not changed since I was pregnant. My husband cooks, washes and cleans and he does it very well (wife B). I did not start doing anything special because of my wife's pregnancy, but I had already washing dishes and cleaning before she was pregnant (husband B).

#### 5.3.3 Category 3: Adequate communication and sharing of thoughts between couples in preparation for parenthood

Five subcategories represented the common perceptions shared between the husband and wife (Table 3). They mutually recognized their adequate communication and sharing of thoughts for the wife's satisfaction. Five examples are given below:

My husband is not at home late at night because of work, so if my labor starts in the evening, he may not be able to come soon. After that, my mother told me that she would come to my house a week before my scheduled birth date (wife G). I am thinking about what to do if something happens to my wife during my work at night. I consulted with her mother so that she could come to our house when it was near the scheduled birth date (husband G).

Although I was not sure whether it would be necessary to work, my husband wants me to work. He worries that I will be alone with our child and isolated from society in the daytime (wife D). Starting from 6 months after the delivery, we will consider whether our child should go to nursery school and whether the wife should work. In the case of my wife, I am anxious about her ability to concentrate on child‐rearing (husband D).

## 6 DISCUSSION

Of the eight women who participated in this study, three were in their 40s. Three women had full‐time jobs. This was higher than the national average employment rate of 26.8% after giving birth to the first child (Japan Ministry of Health, Labor, & Welfare, 2011). Two women had a history of fertility treatment, whereas the rates of complications during pregnancy or delivery and abnormal deliveries were higher than those in standard populations. These findings were considered as characteristic of older primiparous wives.

A careful review of the record unit data revealed that there were no new data, new coding and new categories for targeting eight couples of participants. Therefore, the data have reached saturation as explained in “Analytical methods” section.

### 6.1 The older primiparous wives’ feelings and husbands’ feelings during pregnancy

Two categories were extracted about the couple's feelings during pregnancy. The common and contrasting perceptions within the couple are discussed below.

#### 6.1.1 Mental stress and physical burden associated with older age

In the couples investigated in this study, the wife's perception of the couple involved a feeling of anxiety about the physical burden accompanying her older age and the mental stress caused by minor problems. Generally, older primiparous women are said to be prone to having a higher risk of abnormalities during pregnancy, such as premature birth, and this can cause anxiety. Moreover, after delivery, an older primipara's feeling of fatigue is stronger than that of a multiparous woman; therefore, the risk of postpartum depression is high (Mori et al., 2016; Yamazaki, 2017). The relevance between the physical burdens and depression after birth is clear, but the feeling of mental stress during pregnancy may affect psychological health after birth.

Therefore, it is important to support older women who are concerned about pregnancy and childbirth in ways that allow them to act positively without hesitation while considering personal complications.

#### 6.1.2 Richness and strong will to actively accept the older age

In a previous study that revealed child‐rearing experiences at 1 month after childbirth, older primiparas were generally found to have mental and social strength supported by their career and to take advantage of further increases in self‐growth (Niimura & Ogawa, 2012). In this study, it has become clear that husbands and wives have the advantage of being able to aggressively accept the older, as husbands and wives have various life experiences. An advanced age led to not only uneasiness about the pregnancy, but also a realization about the mental and social advantages, in that they could collaborate to carry out the pregnancy, delivery and child‐rearing.

It has been reported that pregnant older women do not prioritize their career, but rather, temporarily put it on hold and remain more conscious of pregnancy and childbirth (Naota, Miyata, & Okabe, 2001). In the present study, older primiparas made decisions about the continuation of their pregnancy and work like those seen in precedent research. Older pregnant women are expected to have a sense of loneliness, crisis or anxiety because they are forced to become socially isolated by pregnancy, childbirth and childcare. It is possible that older primiparas can make decisions about pregnancy and work adjustments on her own while maintaining a clear mind about her advanced age, thereby enabling the stability of her postnatal mind.

### 6.2 Husbands’ support for their older primiparous wives’ satisfaction during pregnancy

Three categories were extracted about the husbands’ support for the satisfaction of their wives. The common and different perceptions within the couple are discussed below.

#### 6.2.1 Intimacy of the couple

The mental benefits promoted by couple's cooperative actions during pregnancy are an important factor for maintaining the marital relationship; furthermore, these benefits can lead to increased intimacy (Scanzoni & Scanzoni, 1988). The older primiparas in this research had a fear of physical risks and minor problems due to their older age. In this study, older pregnant wives felt satisfied that their husbands were worried about their increased risk of mental burden. Therefore, it is important for the wife to develop marital intimacy to ensure a satisfactory pregnancy and birth. Wives feel satisfaction because their husbands show a greater understanding of the pregnancy and childbirth.

On the other hand, the couple's different perceptions of the support of the satisfied husband of the wife were extracted only to the husband. The husband was worried about the minor problems associated with older primiparous pregnancy and childbirth and even about the physical burden of child‐rearing after birth. However, the wife did not recognize that the husband was worried about these burdens. This suggests a difference in awareness between husbands and wives, a result that was previously reported in a study of primiparous couples. Husband's behaviour/word involvement may be the support of husbands who do not recognize by the wife. A couple's intimacy is signified by the quality of their communication. Intimacy, at its core, is the reaffirmation of each other's presence through words and deeds (Hiraki, 2005). Therefore, it is also important for the wife to express appreciation for her husband's support and to promote sympathy within the couple.

#### 6.2.2 Family system

Within the married life theory of Scanzoni and Scanzoni (1988), the stability of the family system is an essential factor for maintaining a healthy marital relationship. Husbands’ sympathetic support through help with housework also lightens the mood of pregnant wives because of their husbands’ thoughtfulness (Shindo & Wada, 1990). Husbands’ support with household chores can be thought of as a response to their wives’ physical burdens. It is important to maintain the family system and to share the role of housework between husbands and wives. The common perception within couples about wives’ satisfaction of husbands’ support through housework generates a feeling of mutual support.

#### 6.2.3 Consciousness of parenthood

The couple's communication about preparing the child for birth fulfils his wife's expectations for pregnancy and childbirth and develops awareness during pregnancy. This leads to the development of the father's consciousness. If a wife can perceive her husband's consciousness about being a father, this will promote the development of her own consciousness about being a mother.

The older primiparous couples in this research could communicate about the care of the baby after childbirth and adjusting the schedule for a return to the work after the pregnancy. This result differed from previous research before Nakajima and Tokiwa (2011), which clarified the support of the husband who thought that a wife was satisfactory for first pregnant couples. The older primiparous couples were mentally mature, so predicting the changes after childbirth was considered possible during pregnancy. Therefore, it is very important for husbands and wives who are in the process of transitioning to parenthood to prepare for childbirth and child‐rearing through cooperative communication.

## 7 CONCLUSION

1. Older primiparous couples were anxious about pregnancy and childbirth, but they also had mental and social strengths. Thus, it is important for nursing support to understand the feelings of older primiparous couples and to give support that minimizes negative emotions and enhances positive characteristics.
2. There are three concepts of husbands’ support for their older primiparous wives’ satisfaction: the intimacy of the couple, the family system and the consciousness of parenthood. Especially, the development of marital intimacy is considered to lead to good relationships after childbirth. Therefore, nursing support needs to teach partner communication skills to promote understanding of each other's feelings and to be able to adapt to changes in life after childbirth.
3. As a future nursing task, it will be necessary to give a continuous educational program targeting older primiparous couples that encourages a smooth transition from pregnancy to parenthood. It will also be important to give information on the husband's support after childbirth after grasping a better understanding of the mental and social strengths of older primiparous couples.

### 7.1 Limitations

Because we interviewed only eight couples, it is difficult to generalize our results. Accordingly, some perceptions may not have been recorded, even though the couples were actually aware of certain perceptions. Therefore, to obtain a generalizable theory from these data, it is necessary to obtain causal relationships by quantitative research. In addition, future interventional studies of nursing care for enhancing older primiparous wives’ satisfaction towards their husbands’ support should keep in mind the limitations of our research methods.

## CONFLICT OF INTEREST

The authors declare no potential conflicts of interest with respect to the research, authorship and/or publication of this article.

## AUTHOR CONTRIBUTIONS

KN: study design; KN and AU: data analysis; and KN, AU and YH manuscript preparation.

## Supporting information

File S1
